# Treatment outcomes of prolonged induction or re-escalation dosing of upadacitinib in patients with inflammatory bowel disease

**DOI:** 10.1093/crocol/otag021

**Published:** 2026-04-29

**Authors:** Ellen Axenfeld, Vasantham Chaudhary, Joseph W Clinton, Michael Frohlinger, Lauren George, Raymond K Cross, Jordan E Axelrad

**Affiliations:** Department of Medicine, Division of Digestive and Liver Diseases, Columbia University Medical Center, New York, NY, United States; Department of Medicine, Division of Gastroenterology and Hepatology, New York University Langone Health, New York, NY, United States; Department of Medicine, Division of Gastroenterology and Hepatology, University of Maryland School of Medicine, Baltimore, MD, United States; Department of Medicine, University of Maryland School of Medicine, Baltimore, MD, United States; Division of Gastroenterology, MedStar Washington Hospital Center, Washington, DC, United States; Mercy Medical Center, The Center for Inflammatory Bowel and Colorectal Diseases, Baltimore, MD, United States; Department of Medicine, Division of Gastroenterology and Hepatology, New York University Langone Health, New York, NY, United States

**Keywords:** inflammatory bowel disease, upadacitinib, small molecules, dose adjustment

## Abstract

**Background:**

Proposed treatment algorithms favor the use of upadacitinib in medically refractory Crohn’s disease (CD) and ulcerative colitis (UC). There has not yet been data published regarding efficacy of prolonged induction in CD or the efficacy of re-escalation to induction dosing in patients with either CD or UC. The aim of this study was to evaluate the real-world efficacy and safety of prolonged and/or re-escalation upadacitinib therapy in patients with inflammatory bowel disease (IBD) and prior inadequate response to standard upadacitinib treatment.

**Methods:**

This was a retrospective, dual-center study of the efficacy of prolonged induction or re-escalation of upadacitinib in patients with IBD with prior inadequate response to standard upadacitinib treatment.

**Results:**

Fifty-five patients met eligibility criteria. Thirty-nine (70.9%) persisted on upadacitinib therapy while 16 (29.1%) met the primary endpoint for upadacitinib failure. Of those that had comparative objective data, 62.5% had improvement in endoscopic activity, 100% had normalization of an elevated CRP, and 83.3% experienced a decrease in FCP by >50%. There were no new safety signals.

**Conclusion:**

Over two-thirds of patients met the primary endpoint of persistence on therapy without requiring surgery, steroids, or additional biologic/small molecule inhibitor during follow-up. In a subset of patients who had adequate baseline and follow up objective data, the majority of patients had improvement in mucosal healing, decrease by 50% in FCP, and normalization of CRP. This study shows promising results that prolonged upadacitinib induction or dose re-escalation may improve clinical outcomes in a medically refractory patient population.

## Graphical Abstract

**Graphical Abstract:**
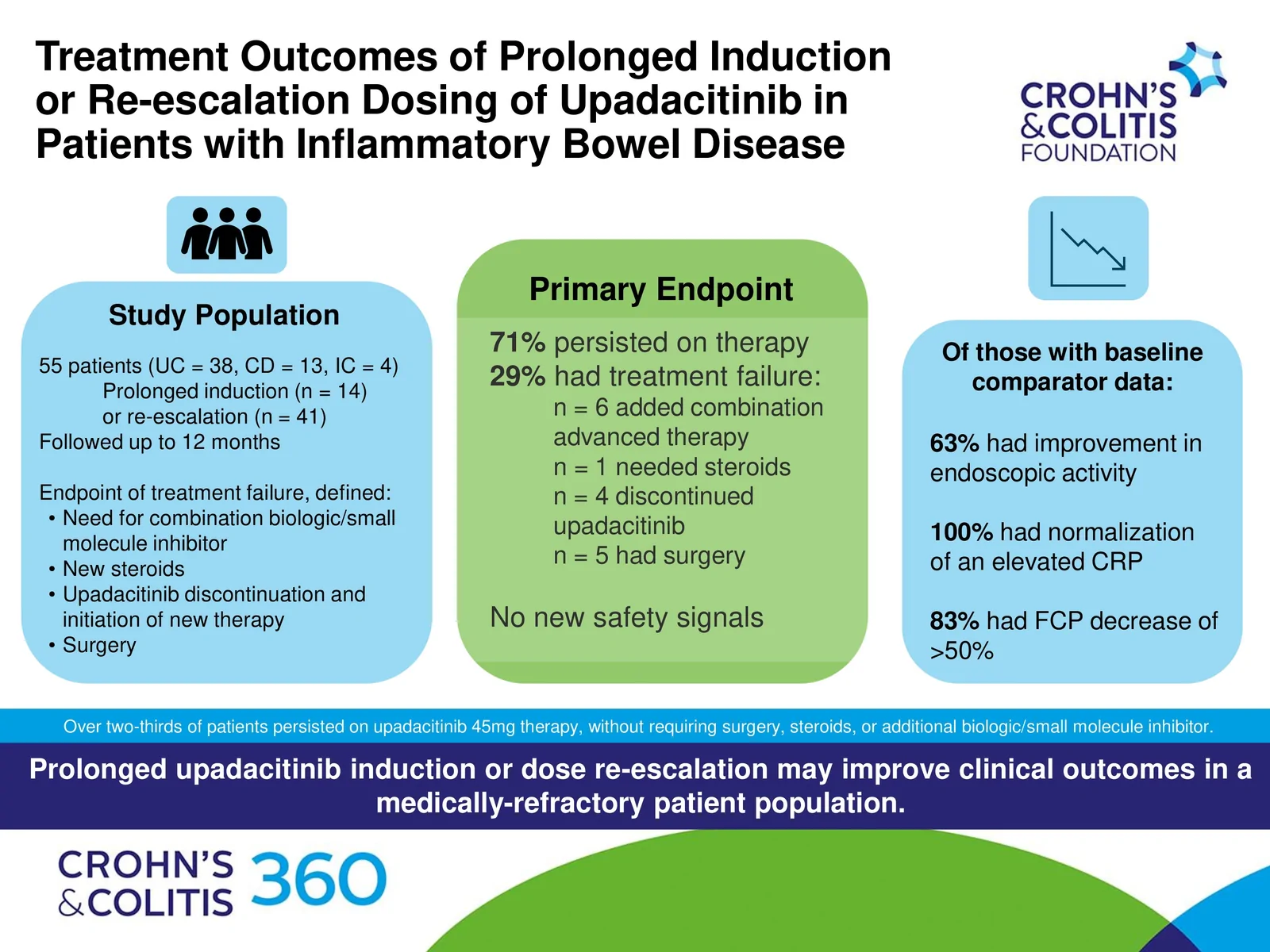

## Introduction

Upadacitinib is an oral Janus kinase (JAK) inhibitor with selective inhibition of JAK1 over JAK2, JAK3, and tyrosine kinase 2. These intracellular tyrosine kinases transduce cytokine-mediated signals via the JAK/STAT pathway and play a key role in immune regulation and inflammation. Dysregulation of this signaling pathway has been implicated in numerous immune-mediated disorders such as IBD, and their inhibition has therefore been shown to be an effective treatment for both ulcerative colitis (UC) and Crohn’s disease (CD).^1^ The selective inhibition of upadacitinib as compared to non-selective JAK inhibitors may allow for higher dosing to achieve more robust efficacy without conceding an increase in safety events.^1^ Proposed treatment algorithms for moderate-to-severe UC and CD favor the use of upadacitinib in medically refractory patient populations, while failure of upadacitinib often results in referral for surgical intervention.^2^ Therefore, options for optimization of medical management with upadacitinib may prove to be a steroid and surgery sparing option.

Standard upadacitinib induction therapy for CD and UC is 45 mg for 12 weeks and 8 weeks, respectively, followed by maintenance dosing with 15 mg or 30 mg based on disease severity. An open-label extension of the U-ACHIEVE trial showed clinical response to extended induction of upadacitinib for 16 weeks in patients with UC who failed to achieve clinical response after the standard 8-week induction period.^3^ No new safety signals were reported in the extension study.^3^ There has not yet been data published regarding the efficacy of prolonged induction in CD as well as the efficacy of re-escalation to induction dosing in patients with either CD or UC. The aim of this study was to evaluate the real-world efficacy and safety of prolonged and/or re-escalation upadacitinib therapy in patients with IBD and prior inadequate response to standard upadacitinib treatment.

## Materials and methods

We performed a dual-center retrospective analysis of clinical outcomes in patients with CD, UC, or inflammatory bowel disease type undetermined (IC) who had inadequate response to standard induction therapy or required re-escalation to induction dosing of upadacitinib between November 2022 and September 2024. The reason for prolonged induction or re-escalation was left to the discretion of the managing provider and was not systematically collected. Baseline data were collected within three months prior to upadacitinib initiation as well as within one month of the start of prolonged induction (ie, 8 weeks after UC induction, 12 weeks after CD induction) or within one month of re-escalation to induction dosing. Follow-up data was considered adequate if collected between one and twelve months after prolonged induction/re-escalation. We began data collection after one month due to the rapid clinical improvement observed in the phase 3 induction studies, which showed clinical improvement within the first week of therapy for both CD and UC.^4^^,^^5^ Collected variables included endoscopic activity, fecal calprotectin (FCP), C-reactive protein (CRP), corticosteroid use, persistence on upadacitinib, addition of a biologic/small molecule, and/or surgical intervention. The primary endpoint was upadacitinib failure after prolonged induction or re-escalation. Failure was defined as the need for a subsequent biologic/small molecule, a new systemic corticosteroid, upadacitinib discontinuation, or surgical intervention. In the patients who persisted on upadacitinib, the baseline (prior to initial upadacitinib induction), pre-escalation (re-escalation or prolonged induction), and follow-up objective data were compared. Endoscopy follow-up data was compared to (1) baseline *or* pre-escalation data, as well as (2) pre-escalation data, alone. FCP and CRP follow-up data was only compared to pre-escalation data.

## Results

Of 723 patients with IBD and a prescription for upadacitinib, 55 met eligibility criteria with adequate follow-up data (UC = 38, CD = 13, IC = 4, Table 1). The majority were white (*n* = 41, 74.5%) and male (*n* = 36, 65.5%). At upadacitinib initiation, the median age was 32 years (interquartile range [IQR] 17.4), and patients had a median IBD duration of 8.7 years (IQR 10.8) with prior exposure to 2.6 (standard deviation [SD] = 1.3) advanced therapies, on average. Of the 55 patients, 41 patients (74.5%) were re-escalated (at an average of 8.2 months after induction, SD = 6.7), and 14 patients (25.5%) required prolonged induction. Over a follow-up period between 1 and 12 months after the date of re-escalation or prolonged induction, 39 patients (70.9%) persisted on upadacitinib therapy, while 16 patients (29.1%) met the primary endpoint criteria for upadacitinib failure. Six patients were prescribed a subsequent biologic or small molecule therapy in combination with upadacitinib, five patients required surgery, four patients discontinued upadacitinib and initiated new therapy, and one required a systemic corticosteroid.

**Table 1:** Characteristics and outcomes of upadacitinib cohort (*n* = 55).

**Demographics**	
** Age, median (IQR)**	32 years (17.4)
** Male, *n* (%)**	36 (65.5)
** White, *n* (%)**	41 (74.5)
** Black, *n* (%)**	4 (7.3)
** Asian, *n* (%)**	2 (3.6)
** Middle Eastern, *n* (%)**	1 (1.8)
** Other/unknown, *n* (%)**	7 (12.7)
**IBD characteristics**	
** Crohn’s disease, *n* (%)**	13 (23.6)
** Ileal**	2
** Colonic**	2
** Ileocolonic**	9
** Ulcerative Colitis, *n* (%)**	38 (69.1)
** Proctosigmoiditis**	2
** Left-sided**	9
** Pancolitis**	27
** Indeterminate colitis, *n* (%)**	4 (7.3)
** Presence of perianal disease, *n* (%)**	6 (10.9)
** Disease duration, median (IQR)**	8.7 years (10.8)
** Number of prior biologic/small molecule exposures, average (SD)**	2.6 (1.3)
**Upadacitinib management**	
** Prolonged induction, *n* (%)**	14 (25.5)
** Re-escalation, *n* (%)**	41 (74.5)
** Time to re-escalation, average (SD)**	8.2 months (6.7)
**Outcomes**	
** Persistence with medication, *n* (%)**	39 (70.9)
** Medication failure, *n* (%)**	16 (29.1)
** Additional biologic/small molecule inhibitor added**	6
** Surgery**	5
** Discontinuation/new therapy started**	4
** Steroid required**	1
** Endoscopic improvement**	
** Baseline *or* pre-escalation to follow-up, *n*/total (%)**	15/15 (100)
** Pre-escalation to follow-up, *n*/total (%)**	5/8 (62.5)
** Composite biomarker (FCP, CRP) improvement, *n* (%)**	10/11 (90.9)

Abbreviations: CD, Crohn’s disease; CRP, C-reactive protein; FCP, fecal calprotectin; IBD, inflammatory bowel disease; IC, indeterminate colitis; IQR, interquartile range; SD, standard deviation; UC, ulcerative colitis.

Fifteen patients had baseline *or* pre-escalation endoscopy reports to compare to follow-up. All 15 (100%) had improvement in the graded activity of their endoscopic evaluation at follow-up. Eight patients had pre-escalation endoscopy reports to compare to follow-up. Of these, five (62.5%) had improvement in endoscopic activity. All eight patients with documented elevated CRP (>5.0 mg/dL) prior to escalation normalized their CRP at follow-up. Only six patients had comparative FCP data, with five experiencing a decrease in FCP by >50%, while one patient had a comparatively unchanged FCP at follow-up. Further exploratory comparisons stratified by patients with upadacitinib persistence versus failure are summarized in Table S1. The only adverse medication effects reported were shingles in one patient and acne in one patient.

## Discussion

In this dual-center study of 55 patients with IBD who required prolonged induction and/or re-escalation of upadacitinib therapy, the majority persisted on therapy without requiring surgery, systemic corticosteroids, and/or additional advanced therapies during follow-up.

The phase 3 trials of upadacitinib in the treatment of both UC (U-ACHIEVE, U-ACCOMPLISH) and CD (U-EXCEL, U-EXCEED, U-ENDURE) showed efficacy over placebo in the induction and maintenance of remission. The subgroup analyses of these trials for both UC and CD demonstrated that upadacitinib maintained significant efficacy over placebo in patients with prior biologic exposure.^4^^,^^5^ With the recent arrival of several novel therapeutics for the treatment of IBD and lack of head-to-head trial data, it is difficult to draw absolute conclusions on the most effective medication for individualized patients, particularly the subset of medically-refractory patients. Prior meta-analyses have attempted to draw these conclusions using the available data. A 2023 meta-analysis of the comparative efficacy of biologic therapies and small molecules in the treatment of UC showed that upadacitinib was the most effective drug with regards to induction of clinical remission and mucosal healing.^6^ Similarly, a 2023 meta-analysis of comparative efficacy of biologic therapies and small molecules in the treatment of luminal CD showed that upadacitinib ranked first for the maintenance of clinical remission.^7^ Not only do proposed treatment algorithms place upadacitinib as a second- and third-line agent, there is also emerging data to support the use of upadacitinib as rescue therapy in acute severe UC.^2^^,^^8^ In the medically refractory IBD patient population, it is important to identify methods to optimize medical therapy, particularly upadacitinib, as it is often a final line of treatment prior to surgery.

In the open-label extension study of U-ACHIEVE, 125 patients with ulcerative colitis received 8 additional weeks of induction therapy with upadacitinib at 45 mg. Of those patients, 48.3% showed a clinical response and were able to then enter the maintenance study.^3^ However, to our knowledge, there has not yet been real-world data published regarding the efficacy of prolonged induction, prior evaluation of this treatment strategy in CD, or the efficacy of re-escalation to induction dosing in patients with either CD or UC.

This study has several strengths. First, it represents the first real-world evaluation of prolonged induction and dose re-escalation strategies with upadacitinib in patients with IBD. The dual-center design increases the generalizability of the findings across different practice settings. Additionally, the inclusion of patients with prior inadequate response to standard induction reflects a highly refractory and clinically relevant population, for whom therapeutic options are often limited. Finally, the favorable safety profile observed in this cohort is reassuring, particularly in a population with cumulative immunosuppression exposure.

However, several limitations must be acknowledged. Most notably, this was a retrospective study with inherent risks of selection bias, missing data, and limited ability to control for confounding variables. Heterogeneity in timing of re-escalation, follow-up intervals, and disease severity at baseline may also have introduced variability in the observed outcomes. Lastly, objective follow-up data were not available for all patients, further limiting definitive conclusions regarding mucosal healing or biochemical response.

Prospective studies are warranted to validate these findings and better define the optimal timing, patient selection, and duration of prolonged or re-escalation dosing strategies. Larger, multicenter cohort studies or registry-based analyses may help overcome sample size limitations and provide more granular insights into predictors of response. Most ideally, randomized controlled trials comparing re-escalation or prolonged induction to continued standard maintenance dosing or alternative therapies would provide high-quality evidence to guide clinical decision-making.

## Conclusion

In our study, we evaluated the clinical outcomes of 55 patients with IBD who received either prolonged induction or re-escalation to induction dosing of upadacitinib. Over two-thirds of these patients met the primary endpoint of persistence on therapy without requiring surgery, steroids, or additional biologic/small molecule inhibitors during follow-up. In a subset of patients who had adequate baseline and follow-up endoscopic data, the majority of patients had improvement in mucosal healing, a decrease by 50% in FCP, and normalization of CRP. Similar to prior reports, there were no new safety signals reported during the follow-up period. This study shows promising results that prolonged upadacitinib induction or dose re-escalation may improve the outcomes of individual patients. Additional and prospective long-term data is required to confirm our findings.

## Supplementary Material

otag021_Supplementary_Data

## Data Availability

The datasets generated during and/or analyzed during the current study are available from the corresponding author on reasonable request.
