# Supplementary material for: Treatment outcomes of prolonged induction or re-escalation dosing of upadacitinib in patients with inflammatory bowel disease
Source: Crohns Colitis 360. 2026 Apr 29;8(2):otag021. doi: 10.1093/crocol/otag021 (PMC13134379; doi:10.1093/crocol/otag021)
Supplement: otag021_Supplementary_Data [file otag021_supplementary_data.docx]

**Supplemental Table 1:** Baseline and outcome characteristics stratified by upadacitinib failure or persistence

|  | **Medication persistence**  ***(n = 39)*** | **Medication failure**  ***(n = 16)*** |
| --- | --- | --- |
| **Gender** | Male = 25 (64%) | Male = 11 (68.8%) |
| **Type of IBD**  CD  UC  IC | 10 (25.6%)  27 (69.2%)  2 (5.1%) | 3 (18.8%)  11 (68.8%)  2 (12.5%) |
| **Number of prior advanced medications - median** | 2 | 2.5 |
| **Medication regimen**  Prolonged induction  Re-escalation | 7 (18%)  32 (82%) | 7 (43.8%)  9 (56.2%) |
| **Baseline endoscopic severity**  Inactive  Mild  Moderate  Severe | 0  2  9  15 | 0  0  3  9 |
| **Baseline CRP (mg/L)** *–* **median (IQR)** | 10.9 (31.9) | 2.0 (23.2) |
| **Baseline FCP (µg/g) – median (IQR)** | 738 (1904) | 1046 (463) |
| **Pre-intervention endoscopic severity**  Inactive  Mild  Moderate  Severe | 0  2  6  5 | 0  1  0  4 |
| **Pre-intervention CRP (mg/L) – median (IQR)** | 2.95 (17.9) | 1.9 (4.5) |
| **Pre-intervention FCP (µg/g) – median (IQR)** | 452 (1313) | 827 (649) |
| **Follow-up endoscopic severity**  Inactive  Mild  Moderate  Severe | 8  7  6  0 | 1  2  2  3 |
| **Follow-up CRP (mg/L) – median (IQR)** | 0 (0.5) | 1.1 (8.3) |
| **Follow-up FCP (µg/g) – median (IQR)** | 234 (255) | 399 (1696) |

Abbreviations: IBD = Inflammatory bowel disease; CD = Crohn’s disease; UC = Ulcerative colitis; IC = Indeterminate colitis; CRP = C-reactive protein; FCP = Fecal calprotectin; IQR = Interquartile range.
